# Distinct Visual Evoked Potential Morphological Patterns for Apparent Motion Processing in School-Aged Children

**DOI:** 10.3389/fnhum.2016.00277

**Published:** 2016-06-28

**Authors:** Julia Campbell, Anu Sharma

**Affiliations:** ^1^Central Sensory Processes Laboratory, Department of Communication Sciences and Disorders, University of Texas at Austin, Austin, TXUSA; ^2^Brain and Behavior Laboratory, Department of Speech, Language and Hearing Science, Institute of Cognitive Science, University of Colorado Boulder, Boulder, COUSA

**Keywords:** high-density EEG, visual evoked potentials, children, sLORETA, source analysis, visual cortical development

## Abstract

Measures of visual cortical development in children demonstrate high variability and inconsistency throughout the literature. This is partly due to the specificity of the visual system in processing certain features. It may then be advantageous to activate multiple cortical pathways in order to observe maturation of coinciding networks. Visual stimuli eliciting the percept of apparent motion and shape change is designed to simultaneously activate both dorsal and ventral visual streams. However, research has shown that such stimuli also elicit variable visual evoked potential (VEP) morphology in children. The aim of this study was to describe developmental changes in VEPs, including morphological patterns, and underlying visual cortical generators, elicited by apparent motion and shape change in school-aged children. Forty-one typically developing children underwent high-density EEG recordings in response to a continuously morphing, radially modulated, circle-star grating. VEPs were then compared across the age groups of 5–7, 8–10, and 11–15 years according to latency and amplitude. Current density reconstructions (CDR) were performed on VEP data in order to observe activated cortical regions. It was found that two distinct VEP morphological patterns occurred in each age group. However, there were no major developmental differences between the age groups according to each pattern. CDR further demonstrated consistent visual generators across age and pattern. These results describe two novel VEP morphological patterns in typically developing children, but with similar underlying cortical sources. The importance of these morphological patterns is discussed in terms of future studies and the investigation of a relationship to visual cognitive performance.

## Introduction

The visual system at the level of the cortex has been described in depth, from single cell recordings demonstrating encoding of object orientation to peripheral deprivation and sensitive periods in visual development (Hubel and Wiesel, 1962, 1970). Overall, the visual network is the most studied and best understood of the sensory systems in the mammalian cortex (Pasternak et al., 2003). In spite of this knowledge, research is ongoing to document the development of the visual system and its many networks across the lifespan. While many studies separate stimulus characteristics in order to evaluate a distinct aspect of the visual system, children develop while perceiving all aspects of visual input simultaneously. Thus, it may be of use to observe visual development when more than one visual network is stimulated. With this in mind, the goal of this study is to describe the development of the visual system and underlying cortical regions in response to transformational apparent motion, which encompasses shape change underlying the perception of motion.

In the cortex, two streams or networks process details of visual stimuli (Van Essen and Maunsell, 1983; Milner and Goodale, 1995). For instance, the dorsal (or ‘where’) pathway consists mainly of magnocellular input and processes motion, while the ventral (or ‘what’) pathway includes both magnocellular and parvocellular input and processes form (Merigan and Maunsell, 1993; Ungerleider and Haxby, 1994; Tootell et al., 1995; Armstrong et al., 2002; Mitchell and Neville, 2004; Bavelier and Hirshorn, 2010). Several neuroimaging studies have found that higher-order visual dorsal regions, including the middle temporal region (hMT+ or V5), are particularly responsive to various types of visual motion, such as moving dots (Culham et al., 2003; Grill-Spector and Malach, 2004; Klaver et al., 2008) while higher-order visual ventral areas, including the fusiform region, respond most strongly when presented with face or object stimuli (Grill-Spector, 2003; Passarotti et al., 2003). As visual complexity increases, *both* the dorsal and ventral visual networks may be responsive to motion and form (Ptito et al., 2003; Tse, 2006).

Continuous morphing of radially modulated visual gratings creates the perception of apparent motion and form change to the viewer, and has been shown to activate higher-order visual cortical regions in V4 and fusiform areas in adults (Allison et al., 1999; Wilkinson et al., 2000; Doucet et al., 2005; Bertrand et al., 2012; Campbell and Sharma, 2014). Area V4 of occipital cortex was initially defined through animal studies in the macaque, and is involved in the processing of motion and shape due to input from both magnocellular and parvocellular pathways (Allison et al., 1984; Gallant et al., 1993, 1996; Ferrera et al., 1994; Tootell et al., 1995). Though less understood in humans, V4 is part of higher-order visual cortex, with feedforward connections directly into inferior temporal cortex and fusiform regions of the ventral visual pathway, critical for facial processing networks (Wilson et al., 1997; Wilkinson et al., 2000; Grill-Spector and Malach, 2004). V4 also appears to coincide regionally with the visual word form area, which is activated during reading tasks and viewing of letter strings (Cohen et al., 2000; Dehaene and Cohen, 2011). With input from both the magnocellular and parvocellular pathways, development of cortical areas comprising V4 may then be indicative of the acquisition of various visual skill sets. Indeed, both magnocellular and parvocellular functional deficits have been found in populations with reading disorders (Li et al., 2012; Gori et al., 2015). Because continuous morphing of radially modulated gratings is thought to excite both magnocellular and parvocellular pathways, it acts as ideal stimuli for the investigation of coinciding visual functional processes, especially those in development prior to the acquisition of certain higher-order skill sets, such as reading.

Little is known regarding the visual cortical development underlying the perception of concurrent apparent motion and shape change. Research using visual evoked potentials (VEPs) via EEG in response to such visual stimuli in children has described developmental changes in peak latency and amplitude, but also identified group and individual differences in the overall morphology of the waveform. This variability in morphology may indicate that visual cortical development, as reflected by changes in the VEP response, should be evaluated not only in terms of peak latency and amplitude, but VEP morphology as well.

Doucet et al. (2005) examined the development of the percept of apparent motion through VEPs recorded in response to the presentation of continuous morphing of radially modulated gratings in children aged 3–22 years. Results showed that while the P1 component of the VEP was stable by age 3 years, N1 and P2 peaks continued to decrease in latency and amplitude through 13 years of age. N1 and P2 VEP peak characteristics are thought to represent the function and neuroplasticity of higher-order visual networks as shown in both adult and child cortical responses to apparent motion, motion, and shape (Taylor et al., 1999; Batty and Taylor, 2002; Mitchell and Neville, 2004; Doucet et al., 2005, 2006; Campbell and Sharma, 2014, 2016). Specifically, in children, it is expected that decreases in latency and amplitude would be observed across age as a result of myelination, synaptic pruning, and strengthened neural networks arising from extrinsic input (Huttenlocher and de Courten, 1987; Taylor et al., 1999; Pallas, 2001; Batty and Taylor, 2002; Mitchell and Neville, 2004; Doucet et al., 2005; Miller et al., 2012). In addition, the authors found a difference in the VEP morphology or overall waveform shape only in the 11–13 years old group. Two morphological subgroups were identified: one with the typical positive-negative-positive (P1-N1-P2) complex, and one with an additional negative-positive peak complex that was not reported to occur in the other age groups. The authors pointed out that one morphological pattern was quite similar to adult-like VEP morphology. Thus, it appears that one group of typically developing children showed a more mature visual cortical response to apparent motion, though reasons for this difference in developmental rates in typically developing children were not discussed. Kubova et al. (2014) also described differential VEP morphology in response to apparent motion in children. The authors reported ‘clear’ and ‘unclear’ VEP morphology elicited by various motion onset stimuli in children aged 7–12 years. ‘Clear’ VEP morphology consisted of an expected positive-negative-positive peak waveform response. VEP responses fell into the ‘unclear’ category if the presence of peak components were difficult to identify and/or consisted of unexpected peaks. For variants of visual motion stimuli, including translation and radial motion, VEP waveforms consisted of expected morphology in 77, 87, and 47% of the children. Furthermore, even clear VEP morphology showed large variance in latency responses. The authors proposed that the reason for the observed variability in VEP morphology in typically developing children may be due to prolonged visual cortical maturation as a result of both intrinsic (e.g., genetic) and extrinsic (e.g., environmental) factors. In any case, it may be useful to categorize VEPs according to both morphology and age in order to more accurately describe visual cortical development in response to apparent motion/shape change.

In the aforementioned study, Doucet et al. (2005) also created topographical scalp maps via VEP peak amplitude to view cortical activation across age in response to apparent motion, but no source localization analyses were conducted to identify specific anatomical generators of the VEP response. These scalp maps showed cortical activation to be stable across age in the occipital region for the VEP P1 peak component, while the N1 and P2 peak components indicated a development change in frontal, temporal, and parietal networks in the processing of apparent motion/shape change. The region of V4 was hypothesized to be largely involved in the processing of apparent motion based upon fMRI research in adults who viewed comparable grating stimuli (Wilkinson et al., 2000). However, identification of V4 as a generator of the VEP response was not possible through the use of topographic scalp maps. Therefore, it remains unclear which cortical networks were activated and may underlie normal differences in visual processing as illustrated by variable VEP morphology, and the manner in which such networks are affected by age.

The overview of the literature above suggests that the latency and amplitude of the distinct morphological changes in VEP response will show systematic age-related changes in school-aged children. These changes should mainly be observed specifically in the N1 and P2 peak components, as reported by Doucet et al. (2005) and driven by neurophysiological changes of increased myelin and synaptic pruning in the school-aged years (Huttenlocher and de Courten, 1987; Pallas, 2001; Miller et al., 2012). In addition, we hypothesize that visual cortical generators of distinct morphological VEP components will show activation in regions approximate to or encompassing V4, in which the response may become more focused as age increases (Wilkinson et al., 2000; Doucet et al., 2005).

Thus, with the goal of examining VEP peak components and identifying underlying visual cortical generators responsive to apparent motion and shape change across the school age years, we recorded high-density EEG while presenting continually morphing, radially modulated, visual gratings in 41 typically developing children over the age range of 5–15 years. We aimed to replicate the morphological patterns described in the aforementioned studies, and if these patterns were indeed observed, to better understand the development of the underlying visual cortical generators.

## Materials and Methods

### Participants

The study included 41 children between the ages of 5 and 15 years. The University of Colorado Institutional Review Board approved the study, and the parents of all participants provided written consent along with the child’s assent. Participants were recruited through advertisements in the community. Parents of participating children reported no hearing loss, no neurological or diagnoses, and normal to corrected vision. Children were grouped in the following age ranges: 5–7 year olds (*n* = 10), 8–10 year olds (*n* = 13), and 11–15 year olds (*n* = 18) consistent with previous VEP developmental studies (Mitchell and Neville, 2004; Doucet et al., 2005) using visual apparent motion and traditional motion stimuli.

### Visual Stimuli

All children were shown a high contrast sinusoidal concentric grating that transitioned into a radially modulated grating or circle-star pattern (Doucet et al., 2005, 2006; see **Supplementary Figure S1**) on a 26-inch flatscreen LCD television at a viewing distance of approximately 42 inches. The circle and star figures were presented 150 times each. The star grating remained on the screen for 600 ms and was immediately followed by the circle grating, also lasting for 600 ms. Observation of the circle-star pattern provided the percept of transformational apparent motion and change in form to the viewer. A total of 300 stimulus presentations (trials) were presented, for a testing time of 3 min. The VEP was time-locked to the onset of each star and circle grating. Participants were instructed to direct their gaze to the center of the pattern at a black dot and to not shift their gaze during the 3 min. All participants reported no difficulty following the instructions.

### EEG Recording and Analyses

A high-density 128-channel EEG electrode recording net (Electrical Geodesics, Inc.) was fitted to the scalp of each participant while they were seated in a comfortable reclining chair in an electro-magnetically shielded sound booth. All stimuli were presented via E-Prime^®^ 2.0, stimulus software compatible with Net Station 4 (Electrical Geodesics, Inc.). Ocular electrodes were designated to record vertical and horizontal eye movements for rejection of ocular artifact. The sampling rate for the EEG recordings was 1 kHz, with an online band-pass filter set at 0.1–200 Hz.

Individual participant EEG data were high-pass filtered oﬄine at 1 Hz and segmented around each stimulus presentation, with 100 ms pre-stimulus and 495 ms post-stimulus time, then exported from Net Station into EEGLAB (Delorme and Makeig, 2004) using MatLab^®^ (The MathWorks^®^, Inc., 2010). The data were baseline-corrected to the pre-stimulus time of -100 to 0 ms, and noisy channels were removed from the recording. Epochs greater than ±100 μV were rejected as artifacts. Data were down-sampled to 250 Hz to reduce processing time, altering the post-stimulus time to 492 ms. Remaining data were re-referenced using common average reference and averaged, and removed channels replaced with interpolated data via a spherical interpolation algorithm.

A central occipital region of interest (ROI) was created for each individual participant by grand-averaging the average VEP response at seven electrodes: 70 or O1, 71, 74, 75 or Oz, 76, 82, and 83 or O2 (Buckley and Tobey, 2011; Campbell and Sharma, 2014, 2016). VEP averages from a central occipital ROI region or electrode have been shown to be sensitive to visual neuroplasticity occurring as a result of age or sensory input (Doucet et al., 2005, 2006; Campbell and Sharma, 2014, 2016). Amplitudes and latencies for individual participants were recorded for all obligatory VEP peaks (i.e., P1, N1, and P2) from the grand VEP average at the central occipital ROI. Individual subject latencies were defined at the highest peak amplitude for each VEP component, or in the midpoint of the peak for broad components. Amplitude was quantified using relative amplitude measures (Gilley et al., 2005; Campbell and Sharma, 2014). P1 amplitude was measured from P1 peak to N1 peak amplitude, N1 amplitude from N1 peak to P2 peak amplitude, and P2 amplitude from P2 peak to the P2 peak offset amplitude. Individual waveform averages were averaged together for each age group to compute a grand-average waveform. VEP waveforms were low-pass filtered oﬄine at 30 Hz for figure presentation purposes only.

### Current Density Reconstruction

Independent component analysis (ICA) was applied to individual participant concatenated EEG data in EEGLAB following artifact rejection and common average referencing (Debener et al., 2006, 2008; Gilley et al., 2008; Campbell and Sharma, 2013, 2014). ICA is a statistical procedure that identifies spatially fixed and temporally independent components that underlie the evoked potential (Makeig et al., 1997), and is used to successfully model cortical EEG sources (Makeig et al., 1997, 2004; Hine and Debener, 2007; Debener et al., 2008; Gilley et al., 2008; Campbell and Sharma, 2013, 2014, 2016). Independent components representing artifact, such as eye blinks and line noise that were not rejected in the initial EEG artifact rejection, were first removed from the data. Next, the underlying independent components that accounted for greatest percent variance of the VEP peaks (e.g., P1, N1, and P2) were identified and exported into CURRY^®^ Scan 7 Neuroimaging Suite (Compumedics Neuroscan^TM^) for source modeling. In CURRY, the selected components were averaged into the appropriate age groups according to each VEP peak (e.g., all P1 components in 5–7 year olds), and an additional ICA was run for each group for verification of relevant independent components.

Current density reconstructions (CDR) were then performed for each VEP component of each age group, using sLORETA (standardized low-resolution brain electromagnetic tomography). sLORETA is a specific statistical method that takes into account variance of the cortical source itself as well as variance arising from EEG measurement noise (Pascual-Marqui, 2002; Grech et al., 2008). Head models appropriate for the age groups were created using Boundary Element Method (BEM) geometry (Fuchs et al., 2002) in CURRY based upon developmental white matter averages provided by Wilke et al. (2007) and Gilley et al. (2008). Resulting group CDRs were represented by a graded color scale image placed on a Montreal Neurological Institute (MNI) child MRI provided in CURRY. Sagittal MRI slices were selected to illustrate the greatest differences in cortical activation between the groups.

## Results

### Visual Evoked Potentials

**FIGURE 1:**
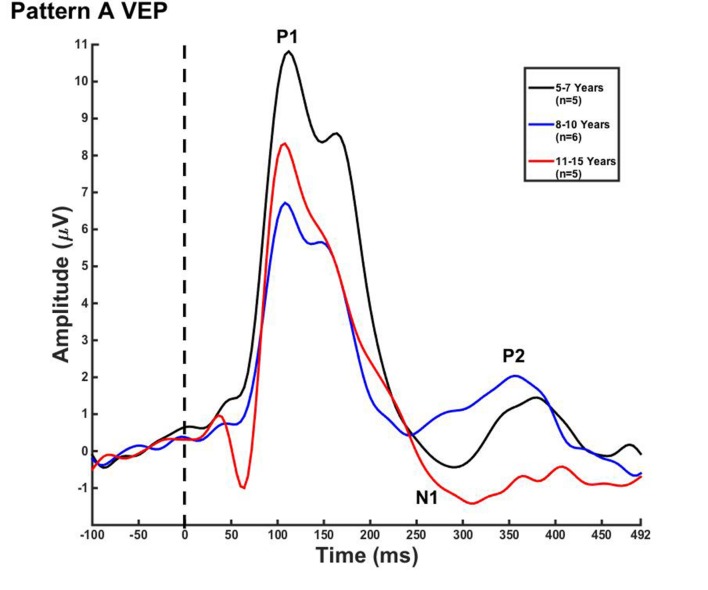
**Visual evoked potential (VEP) pattern A in children 5–7, 8–10, and 11–15 years old.** VEP waveforms from the occipital region of interest (ROI) in 5–7 year olds (black), 8–10 year olds (blue), and 11–15 year olds (red). Amplitude is depicted on the vertical axis in microvolts and latency on the horizontal axis in milliseconds. The legend in the upper right shows the age groups according to waveform color and number of subjects.

**FIGURE 2:**
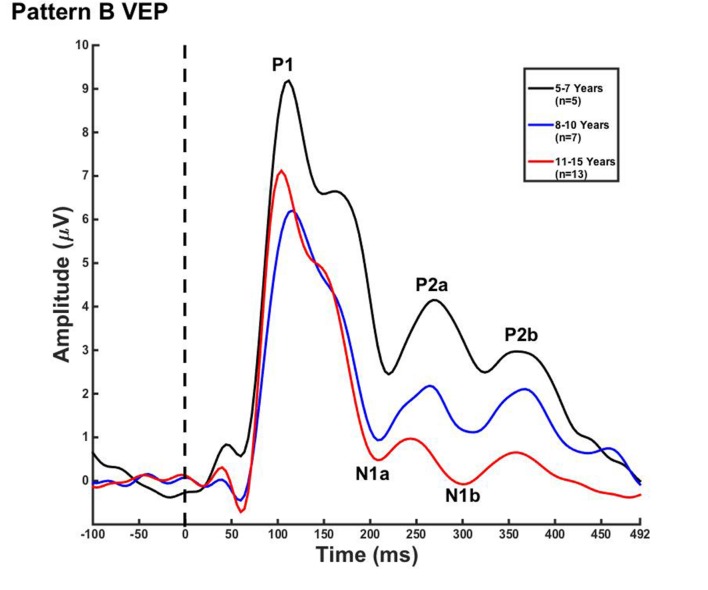
**Visual evoked potential pattern B in children 5–7, 8–10, and 11–15 years old.** VEP waveforms from the occipital region of interest (ROI) in 5–7 year olds (black), 8–10 year olds (blue), and 11–15 year olds (red). Amplitude is depicted on the vertical axis in microvolts and latency on the horizontal axis in milliseconds. The legend in the upper right shows the age groups according to waveform color and number of subjects.

Visual evoked potential analyses revealed two clear morphological patterns for each age group. Children who showed a VEP response consisting of three obligatory peaks (P1, N1, and P2) were classified as pattern A (**Table 1**; **Figure 1**). Children with a VEP response consisting of multiple peaks (P1, N1a, P2a, N1b, and P2b) were classified as pattern B (**Table 2**; **Figure 2**). Peak components for pattern A were identified as follows: P1 as the first positive peak occurring at approximately 100 ms, N1 as the first negative peak at approximately 270 ms, and P2 as the second positive peak occurring at approximately 360 ms. Peak components for pattern B were identified as follows: P1 as the first positive peak occurring at approximately 100 ms, N1a as the first negative peak occurring at approximately 200 ms, P2a as the second positive peak occurring at approximately 250 ms, N1b as the second negative peak occurring at approximately 300 ms, and P2b as the third positive peak at approximately 360 ms. **Figures 1** and **2** show the average VEP waveforms for the 5–7, 8–10, and 11–15 year olds that were categorized as pattern A and B, respectively. For the 5–7 years old group, five children (50%) demonstrated pattern A morphology, and 5 children (50%) showed pattern B morphology. In the 8–10 years old group, six children (46%) showed pattern A morphology, while seven children (54%) exhibited pattern B morphology. Finally, five children (28%) in the 11–15 years old group showed pattern A morphology, while the majority of 13 children (72%) showed pattern B morphology. There was no significant difference in ages of children exhibiting pattern A (*n* = 16, mean age and standard deviation = 9.90 ± 2.33 years, range = 7.02–13.99 years) vs. pattern B (*n* = 25, mean age and standard deviation = 10.58 ± 2.40 years, range = 5.87–14.00 years) [*t*(39) = -0.891, *p* > 0.05]. Comparable morphological patterns have been observed in response to a similar visual stimulus (Doucet et al., 2005). See **Tables 1** and **2** for the mean values of the VEP peak components in patterns A and B, respectively.

**Table 1:** Visual evoked potential (VEP) pattern A data in children 5–15 years old.

	Age	P1 Latency	P1 Amplitude	N1 Latency	N1 Amplitude	P2 Latency	P2 Amplitude
**5–7 year olds (*n* = 5)**							
Mean	7.33	106.4	12.08	276.8	3.49	380	2.51
Standard deviation	0.27	7.27	2.28	39.33	1.95	20.59	0.94
**8–10 year olds (*n* = 6)**							
Mean	9.7	106	6.83	241.33	2.73	327.33	2.47
Standard deviation	1.06	4.9	1.76	16.72	1.27	56.25	1.22
**11–15 year olds (*n* = 5)**							
Mean	12.72	108	10.21	301.6	2.04	389.6	2.11
Standard deviation	0.83	2.83	3.49	30.01	0.88	27.22	1.23
**VEP pattern A Group total**							
Mean	9.9	106.75	9.53	271.25	2.75	363.25	2.37
Standard deviation	2.33	5	3.3	37.7	1.45	46.97	1.08

*VEP peak component latency and amplitude means and standard deviations are provided for each age group presenting with VEP pattern A. The mean and standard deviations for peak components combined across all ages is shown in the last two rows. Latency is in milliseconds and amplitude is in microvolts.*

**Table 2:** Visual evoked potential (VEP) pattern B data in children 5–15 years old.

	Age	P1 Latency	P1 Amplitude	N1a Latency	N1a Amplitude	P2a Latency	P2a Amplitude	N1b Latency	N1b Amplitude	P2a Latency	P2b Amplitude
**5–7 year olds (*n* = 5)**											
Mean	6.89	119.20	6.79	208.80	2.73	258.40	2.66	307.20	2.02	352.80	3.41
Standard deviation	0.60	18.42	2.50	33.27	1.61	39.46	1.03	47.62	1.39	51.02	1.60
**8–10 year olds (*n* = 7)**											
Mean	9.72	112.57	6.06	204.57	1.83	245.14	1.91	297.71	1.91	377.71	2.68
Standard deviation	0.94	10.94	3.01	28.88	1.73	33.68	1.43	57.26	0.93	24.96	2.36
**11–15 year olds (*n* = 13)**											
Mean	12.47	104.92	7.29	203.08	1.64	242.77	2.49	300.00	2.22	366.46	2.06
Standard deviation	1.02	6.56	3.33	23.95	1.35	26.50	1.68	38.61	1.38	36.90	1.64
**VEP pattern B Group total**											
Mean	10.58	109.92	6.84	204.64	1.91	246.56	2.36	300.80	2.09	366.88	2.50
Standard deviation	2.40	11.90	3.02	26.17	1.51	30.53	1.48	44.21	1.23	36.69	1.86

*VEP peak component latency and amplitude means and standard deviations are provided for each age group presenting with VEP pattern B. The mean and standard deviations for peak components combined across all ages is shown in the last two rows. Latency is in milliseconds and amplitude is in microvolts.*

For each VEP pattern, latencies and amplitudes were compared across age using the Kruskal–Wallis test. When determining significance between the three age groups, *post hoc* pairwise comparisons were calculated using the Bonferroni correction for multiple comparisons. In pattern A (**Figure 1**), two significant developmental differences were observed. First, the 5–7 years old group showed significantly larger P1 amplitude than the 8–10 years old group [χ^2^(2) = 2.798, *p* = 0.015]. This finding is consistent with previous reports of age-related decreases in VEP amplitude (Snyder et al., 1981; Allison et al., 1984; Mitchell and Neville, 2004; Doucet et al., 2005; Mahajan and McArthur, 2012). In addition, the 8–10 years old group demonstrated a significantly earlier N1 latency than the 11–15 year olds [χ^2^(2) = -2.594, *p* = 0.028]. This finding is atypical, as cortical evoked potential latency normally decreases with cortical maturation or increasing age (Madrid and Crognale, 2000; Mitchell and Neville, 2004; Doucet et al., 2005). In contrast to pattern A, pattern B (**Figure 2**) did not show any significant latency or amplitude differences across age. This result is consistent with other developmental findings in vision that have observed relatively stable VEP latency across age (Mahajan and McArthur, 2012).

**FIGURE 3:**
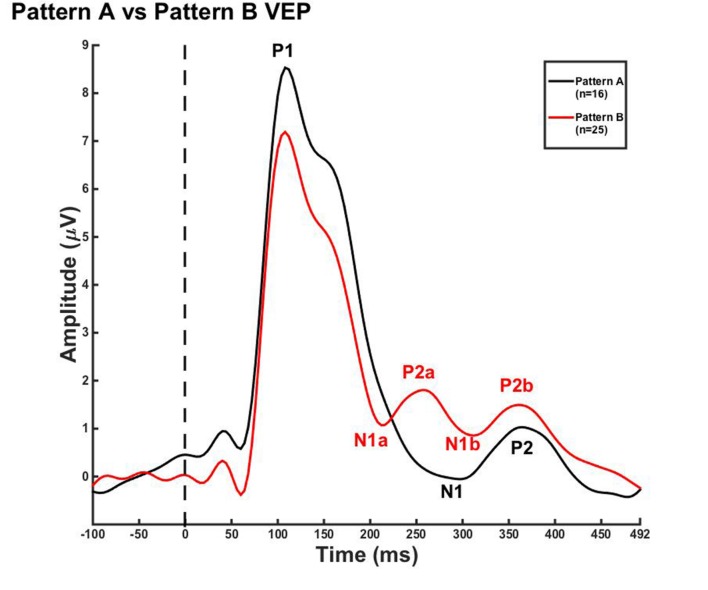
**Visual evoked potential patterns A and B in children 5–15 years old.** VEP waveforms from the occipital region of interest (ROI) for pattern A (black) and pattern B (red). Amplitude is depicted on the vertical axis in microvolts and latency on the horizontal axis in milliseconds. The legend in the upper right shows the age groups according to waveform color and number of subjects.

Due to the lack of major developmental differences between the age groups for each pattern, the average VEP waveforms were collapsed across age groups in order to provide a grand average waveform comparison between pattern A and pattern B (**Figure 3**). Statistical comparisons were made using the Kruskal–Wallis test. The Bonferroni correction for multiple comparisons was applied for *post hoc* pairwise comparisons. As seen in **Figure 3**, P1 amplitude was significantly greater in pattern A compared to pattern B [χ^2^(1) = 5.915, *p* = 0.015], while both N1a and P2a peak components occurred at significantly earlier latencies in pattern B in comparison to N1 and P2 peak components in pattern A [χ^2^(2) = 4.026, *p* = 0.000; χ^2^(2) = 5.363, *p* = 0.000]. In contrast, there were no significant differences between P2b and P2 and N1b and N1 (*p* > 0.05). Thus, the data in **Figure 3** clearly illustrate that N1a and P2a appear as additional independent components in pattern B.

### Current Density Reconstruction

**FIGURE 4:**
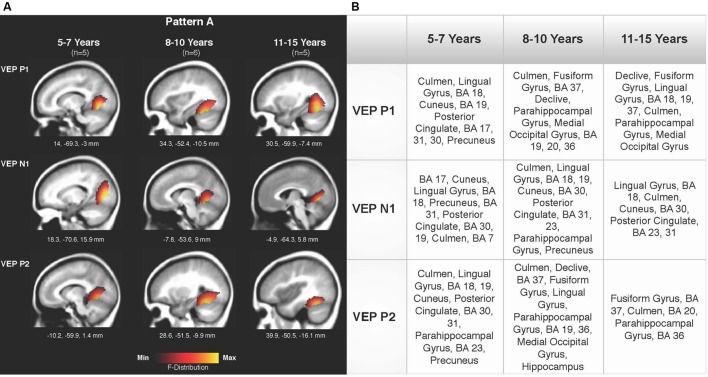
**Current density reconstructions (CDR) for VEP pattern A in children 5–7, 8–10, and 11–15 years old. (A)** Cortical activations for each VEP peak component (P1, N1, and P2) in Pattern A are shown for the 5–7, 8–10, and 11–15 years old groups. CDR images are presented on sagittal MRI slices, with three-dimensional Montreal Neurological Institute (MNI) coordinates beneath each slice. The *F*-Distribution scale at the bottom of the figure illustrates the range of highest (yellow) and lowest (red) activation. **(B)** A table listing responsive cortical regions in order of highest to lowest activation for each VEP component in each age group.

**FIGURE 5:**
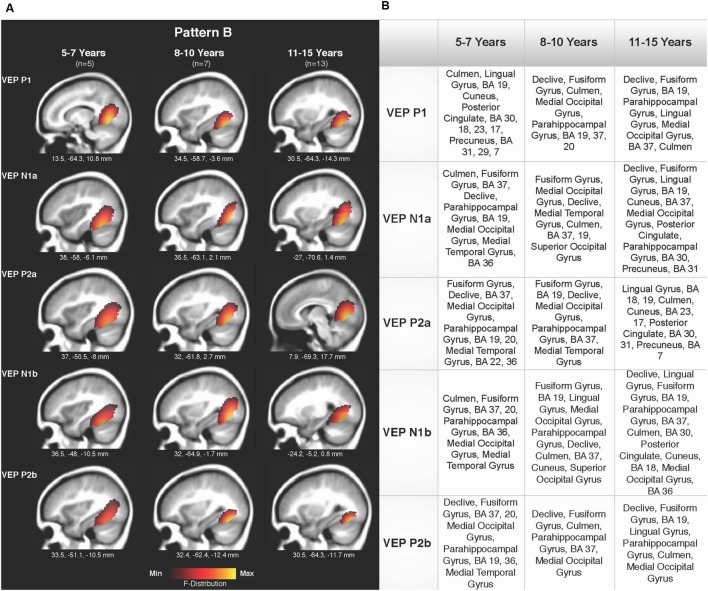
**Current density reconstructions for VEP pattern B in children 5–7, 8–10, and 11–15 years old. (A)** Cortical activations for each VEP peak component (P1, N1, and P2) in pattern B are shown for the 5–7, 8–10, and 11–15 years old groups. CDR images are presented on sagittal MRI slices, with three-dimensional MNI coordinates beneath each slice. The *F*-Distribution scale at the bottom of the figure illustrates the range of highest (yellow) and lowest (red) activation. **(B)** A table listing responsive cortical regions in order of highest to lowest activation for each VEP component in each age group.

**FIGURE 6:**
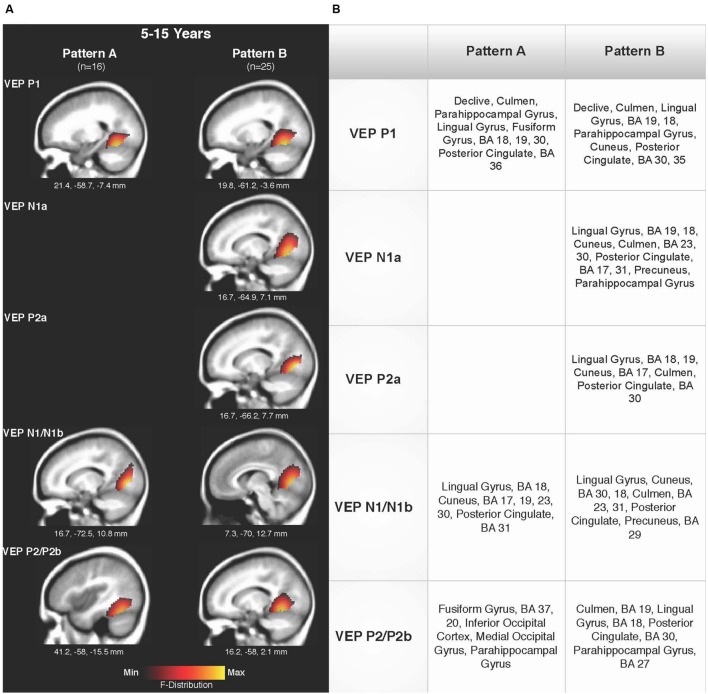
**Current density reconstructions for VEP patterns A and B in children 5–15 years old. (A)** Cortical activations for each VEP peak component averaged across age in pattern A (P1, N1, and P2) and Pattern B (P1, N1a, P2a, N1b, and P2b) are shown. CDR images are presented on sagittal MRI slices, with three-dimensional MNI coordinates beneath each slice. The *F*-Distribution scale at the bottom of the figure illustrates the range of highest (yellow) and lowest (red) activation. **(B)** A table listing responsive cortical regions in order of highest to lowest activation for each VEP component in each age group.

Current density reconstructions were calculated using the sLORETA algorithm provided by CURRY^®^ Scan 7 Neuroimaging Suite. CDR images were created using independent components accounting for VEP peaks in each age group for patterns A (**Figure 4**) and B (**Figure 5**), as well as the collapsed pattern A and B comparison (**Figure 6**). The resulting activations were superimposed on an average MRI (sagittal slice view) with the MNI co-ordinates shown beneath each slice. The scale of the F distribution, indicating the strength of the activations, is also shown. Activated cortical regions in approximate order of response strength are listed in the table to the right of the CDR images.

**Figures 4A,B** depicts the CDR images for VEP P1, N1, and P2 components in the 5–7, 8–10, and 11–15 years old groups for pattern A. Overall, visual cortical generators were comparable between the age groups, with expected activation of cerebellar areas, striate, and extrastriate visual cortex including Brodmann Areas 18, 19, fusiform gyrus, and lingual gyrus (Bucher et al., 2006; Klaver et al., 2008; Campbell and Sharma, 2014, 2016). The CDR images for the 5–7 years old group suggest a possibly less mature cortical response in comparison to the older age groups since activation of the fusiform gyrus was only present for the P1 component. The fusiform gyrus and ventral visual pathway have been shown to be largely responsive to modulated visual gratings in adults (Wilkinson et al., 2000; Bertrand et al., 2012; Campbell and Sharma, 2014), with cerebellar regions also indicated to be involved in processing complex visual stimuli (Dupont et al., 2003; Kellermann et al., 2012; Campbell and Sharma, 2014).

Pattern B CDR images for P1, N1a, P2a, N1b, and P2b are shown in **Figures 5A,B** for the 5–7, 8–10, and 11–15 years old groups. As observed for pattern A, overall cortical activation was consistent in striate and extrastriate areas across components and age groups. Main areas of activation included Brodmann Areas 18 and 19, fusiform gyrus, lingual gyrus, and cerebellar areas. Similar to pattern A, pattern B depicted a different response than that of adults to modulated visual gratings, with a more diffuse source that approached but is not completely localized to fusiform and cerebellar regions (Wilkinson et al., 2000; Bertrand et al., 2012; Campbell and Sharma, 2014).

**Figures 6A,B** shows the CDR results for pattern A collapsed across all age groups (5–15 years), and pattern B collapsed across all age groups. This comparison allowed for observation of possible different activations of visual cortex between the two patterns. Consistent with the previously described CDR results for both patterns, cortical activation was present in areas comprising Brodmann Areas 18 and 19, fusiform gyrus, lingual gyrus, and cerebellum. The P1 and P2 components in Pattern A, as well as the P1 and P2b components in pattern B, showed more fusiform and cerebellar activation, approaching that of the adult response to similar stimuli (Wilkinson et al., 2000; Bertrand et al., 2012). Meanwhile, the N1 component of pattern A and N1a, P2a, and N1b components of pattern B were more concentrated in medial occipital regions involving striate visual areas. In addition, it appears that pattern B revealed a visual network with multiple ‘steps’ for the percept of apparent motion and shape change in comparison to pattern A, as independent components (N1a, P2a) reflected two additional regions of activity in the visual cortex encompassing cuneus, lingual gyrus, cerebellum, and Broadmann Areas 17, 18, and 19. Aside from this difference, as can be seen in **Figure 6**, there did not appear to be any other major difference between responsive visual cortical areas underlying VEP components in pattern A and B.

## Discussion

The goal of this study was to examine cortical development of higher-order visual processing in typically developing children using an apparent motion stimulus (i.e., a continuously morphing, radially modulated, circle-star grating; see **Figure 1**). We recorded high-density EEG in 41 typically developing children and compared VEP peak components and morphological patterns across children 5–7, 8–10, and 11–15 years of age (Mitchell and Neville, 2004; Doucet et al., 2005). Additionally, CDR images were created using independent VEP components in order to view activation of cortical regions elicited by the stimuli as a function of age and VEP morphology.

The main findings of this study are as follows: (i) Two VEP morphological patterns were identified in each age group. Pattern A consisted of the classic VEP response morphology, comprised of three obligatory peaks (P1, N1, and P2; **Figure 1**). Pattern B included additional peak components (P1, N1a, N1b, P2a, and P2b; **Figure 2**). Interestingly, there were no major developmental differences across age for either pattern, which allowed for the averaging of patterns A and B across age for the CDR analyses. (ii) CDR imagery illustrated the active cortical generators underlying each VEP peak component, consistently showing responses in lingual gyrus, cerebellum, and Broadmann Areas 18 and 19. Visual generators were robust and stable across age and components for both patterns A (**Figure 4**) and B (**Figure 5**). When all age groups were combined according to pattern (**Figure 6**), visual cortical activation remained consistent, though pattern B clearly demonstrated redundant or additional activations of visual cortex.

### Morphological Patterns

Two morphological patterns were elicited by apparent motion/shape change. Pattern A consisted of the typical P1, N1, P2 peak response, while pattern B consisted of a multi-peak waveform comprised of P1, N1a, P2a, N1b, and P2b components. VEP morphology similar to that of pattern B has been described by Doucet et al. (2005) in children viewing comparable modulated grating stimuli. However, Doucet et al. (2005) only identified morphology similar to pattern B in children between the ages of 11–13 years, though subjects as young as 3 years were included in the study. Thus, the authors hypothesized that a multi-peak VEP response may be indicative of more adult-like processing of apparent motion and shape change. In contrast, our results show pattern B in children as young as 5–7 years. This discrepancy between the studies may be due to the amount of presentation trials collected in each study. For example, approximately 50 trials are considered sufficient for a valid VEP recording (Kremlacek et al., 2004; Doucet et al., 2005; Kubova et al., 2014). However, because of CDR analyses and the importance of optimizing signal-to-noise ratio of the components (Campbell and Sharma, 2013, 2014, 2016), we recorded well over 150 trials for each individual. Inclusion of these additional trials may have improved the signal-to-noise ratio allowing for enhanced visualization of additional peak components. Consistent with the results of the present study, multi-peak VEP waveforms have been described in other developmental studies using comparable stimuli, and observed in children as young as 5 years (Moskowitz and Sokol, 1983; Kubova et al., 2014). Thus, there appears to be identifiable individual variability in VEPs reflective of visual cortical processing. At present, it is unclear whether normal individual variability in VEP morphological patterns, or these two specific patterns, are indicative of visual behavioral performance, cognition, or psychological traits in school-aged children. Behavioral cognitive measures could show a relationship between these networks and variations in visual learning and development, especially if examined in the time-frequency domain (Basar et al., 2001; Posner and Rothbart, 2005; Harmony, 2013). For instance, VEP patterns A and B suggest that there are at least two visual cortical networks in typically developing children related to the percept of apparent motion/shape change. These networks appear to differ in temporal processing (i.e., one network shows two extra processing ‘steps’) rather than in location of cortical generators. Because these networks differ in the temporal domain, time-frequency analyses may shed light on underlying frequency oscillations which modify the VEP response. For example, Gould et al. (2011) found significant changes in the alpha frequency band (8–12 Hz) recorded via EEG during a visuospatial attention task. During this task, participants were provided with a cue prior to the presentation of a target. As spatial certainty increased, alpha power decreased. In other words, the alpha frequency band was related to ‘anticipation’ of the target, which predicted reaction times and VEP P1-N1 amplitude. Similarly, Hanslmayr et al. (2007) presented visual letter stimuli of short duration to participants while recording EEG. Participants were asked to determine which of four letters that they perceived. It was found that for the group who successfully perceived the letters, pre-stimulus alpha band power was significantly lower than for those who were not able to perceive the letters. This result illustrates that on-going EEG (i.e., not necessarily elicited by stimuli) may have a significant effect not only on the morphology of evoked potential responses, but visual perception as a whole (Mathewson et al., 2009). Furthermore, phase coupling in the beta-gamma frequency ranges (20–45 Hz) was also related to successful perception of the visual letters (Hanslmayr et al., 2007). Both studies illustrated that alpha band power was related to individual variability in behavioral performance. Though the task in our experiment did not involve active participation, but was passive, the robust morphological difference in the VEP responses does point to a distinction in the cortical oscillations of the children. Indeed, some children may have been anticipating the shape change underlying the apparent motion percept or were more attentive to the stimuli in general (Zhang et al., 2013). In general, research has demonstrated that cortical frequency oscillations may underlie normal variability in visual perception. As a result, we plan to further examine a possible functional relationship between VEP morphology, visual cortical oscillations, and cognitive performance in typically developing children. In the future, we plan to apply relevant findings to clinical populations, especially as VEP responses have shown to be affected in dyslexia, psychological disorders, learning disorders, and various neurocognitive disorders (Conners, 1970; Lehmkuhle, 1993; Fotiou et al., 2003; Mitchell and Neville, 2004).

### Visual Evoked Potentials

In the current study, we observed few developmental changes in VEP peak latencies and amplitudes. P1 amplitude was significantly larger for the 5–7 year olds, and N1 latency was significantly earlier in the 8–10 year olds in pattern A (**Figure 1**). In pattern B, no significant amplitude or latency differences were found (**Figure 2**). The lack of major developmental differences in P1 peak amplitude and latency is expected and consistent with other studies that have shown stability of this component as early as age 3 years, and as it is considered to arise from primary visual cortex (Moskowitz and Sokol, 1983; Doucet et al., 2005; Whittingstall et al., 2007). Neuroimaging data have determined that primary cortical areas develop first, and must mature prior to secondary cortices, offering a possible explanation as to why the VEP P1 is extremely stable early in life and for a variety of stimuli (Gogtay et al., 2004). However, latency and amplitude of the later VEP peaks have been found to be highly variable and dependent upon certain aspects of visual stimuli, such as motion type and pattern size (Moskowitz and Sokol, 1983; Kremlacek et al., 2004; Bucher et al., 2006; Lefebvre et al., 2009; Kubova et al., 2014) as well as the portion of visual cortex or visual pathway that is being activated (Allison et al., 1984; Mitchell and Neville, 2004). For example, Mahajan and McArthur (2012) presented pattern reversal checkerboard stimuli to adolescents aged 10–18 years. Though amplitude changes were observed across age, no consistent changes in latency of VEP components were seen. In contrast, Allison et al. (1984) described decreased VEP component latency in response to checkerboard reversal patterns into middle age. The results of these studies illustrate the high variability of latency and amplitude changes across development in the VEP response in children, suggesting that grouping response waveforms according to morphological patterns (as we report in the present study) may allow for greater accuracy in observing systematic evoked potential changes in visual cortical maturation.

Despite comparing VEP responses across age according to similar morphology, our findings showed that neither VEP pattern A or B presented major maturational changes as reflected by amplitude and latency across the age range of 5–15 years. However, it is important to note that VEP pattern A, especially in terms of latency for the N1 and P2 peaks, is not equivalent to that of adult VEP responses to similar gradient stimuli (Doucet et al., 2005, 2006; Campbell and Sharma, 2014). In contrast, the N1a and P2a peaks of pattern B are more similar to the adult VEP N1 and P2, though overall morphology is dissimilar to the adult VEP considering the additional peaks of N1b and P2b. These differences indicate that significant maturation of higher-order visual cortex underlying the processing of motion/form stimuli may take place after age 15 years. Indeed, Allison et al. (1984) have described VEP latencies in response to checkerboard patterns to decrease well into the third decade of life. Furthermore, future analyses in the time-frequency domain of variable VEP morphology as a result of apparent motion stimuli may illustrate developmental changes that were not observed in this study (Uhlhaas et al., 2010). Additional research is necessary to determine at what point visual processing for apparent motion stimuli becomes adult-like.

### Current Density Reconstruction

Visual cortical activation in children in response to radially modulated gratings was quite similar across patterns when evaluated according to age groups (**Figures 4** and **6**) and when patterns A and B were compared (**Figure 6**), with consistent responses in lingual gyrus, cerebellum, and Brodmann Areas 18 and 19. CDR images for patterns A and B illustrated the lack of difference in major visual network activation across age (**Figures 4** and **5**). There was a slight developmental shift in cortical response for pattern A, as the response of the 5–7 year olds was oriented medially and superiorly in comparison to the stronger ventral response of the 8–10 and 11–15 year olds (**Figure 4**). In contrast, the visual generators illustrated in pattern B were comparable for each age group, across components (**Figure 5**).

Because of the similarities in activated cortical regions, age groups within each pattern were combined and CDRs calculated for the VEP components. When age groups were combined within each morphological pattern, these similarities remained robust, with all components showing activation in striate and extrastriate regions (**Figure 6**). Though feedforward and feedback networks between frontal and occipital cortices have been identified for various visual functions (Pantazatos et al., 2012), we did not observe a response in such networks to apparent motion. This lack of frontal activation may be related to the passivity of the task. For both VEP patterns, visual cortical activation underlying P1 and P2/P2b VEP components tended to be more ventral in location, while N1, N1a, P2a, and N1b components reflected a trend toward more superior and posterior occipital activation (**Figure 6**). This slight shift in the visual response may represent feedback and feedforward communication between primary and higher-order visual areas underlying processing and awareness of complex visual input (Lamme, 2001; Silvanto et al., 2005). In addition, pattern B VEP morphology represented additional ‘steps’ in apparent motion processing in children, with two additional components (N1a and P2a) reflective of visual cortical function. Again, it will be an important next step to investigate whether cortical frequency oscillations are modulating the VEP response to apparent motion. If so, it will be of interest to evaluate the cortical source generators of such oscillatory networks and the functional relevance of these networks in relation to apparent motion processing in children. Cortical networks involved in apparent motion processing, including magnocellular and parvocellular function, have been implicated in developmental reading disorders (Pugh et al., 2001; Englund and Palomares, 2012). Overall, these cortical responses observed in this study are consistent with imaging studies in children showing responses in the fusiform region, lingual gyrus, and cuneus in response to faces, motion-defined form, and reading (Passarotti et al., 2003; Shaywitz and Shaywitz, 2005; Bucher et al., 2006; Meschyan and Hernandez, 2006).

Altogether, in our study, the responsive visual cortical regions in children appeared to be different from those reported in adult studies. For instance, we did not observe activation of typical visual motion generators, such as hMT+/V5 in the dorsal pathway (Paradis et al., 2000; Donner et al., 2007). However, it is thought that the percept of apparent motion and shape change, similar to that presented in this study, involves both magnocellular and parvocellular pathways (Gallant et al., 1993, 1996; Ferrera et al., 1994; Tootell et al., 1995) and is processed differently from ‘true’ visual motion (Bertrand et al., 2012). Adult studies, which have presented visual stimuli consistent with ours, have described greater ventral activation involving V4 (which receives input from both magnocellular and parvocellular networks) and fusiform areas (Allison et al., 1999; Wilkinson et al., 2000; Bertrand et al., 2012). For instance, Allison et al. (1999) performed intracranial recordings in seizure patients in response to static visual gratings. Subsequent activation observed in V4 was posterior to the responses observed in this study. These discrepancies in regional activation observed between our study and the results reported by Allison et al. (1999) may indicate on-going development of V4 in children, with possibly increasing activation in adult V4 areas as age increases. There may also be additional stimuli differences to consider as Allison et al. (1999) presented static radial gratings that did not include apparent motion (Bertrand et al., 2012). Taking these findings into consideration, it is possible that the visual cortical response observed in the children in this study may become more adult-like as age increases, though at what age this change would occur, or what type of visual skill development may be related, is presently unknown.

Overall, there were no major differences across age for VEP morphology, component latency or amplitude, or in activated visual cortical regions as illustrated by CDR imagery. However, two distinct VEP morphological patterns were observed, which to our knowledge have not been previously described to such an extent (Doucet et al., 2005; Kubova et al., 2014). The underlying visual cortical networks involved in the processing of continuously morphing, radially modulated stimuli appear to be remarkably robust and similar, despite the distinct morphological differences in the VEP response waveforms. Recent studies from our laboratory (Sharma et al., 2014; Campbell and Sharma, 2016), consistent with previous studies (Doucet et al., 2005, 2006), suggest that the visual apparent motion stimuli described in this study in combination with source localization are indeed useful for the study of visual plasticity. For example, we have recently reported that the visual stimuli described in the present manuscript elicited activation of auditory cortical areas (suggestive of cross-modal re-organization) in a deaf child who was an average cochlear implant user, while only visual areas were activated in a child who was good cochlear implant user (Sharma et al., 2014). These findings illustrate the sensitivity of these stimuli in evaluating visual plasticity in typically developing children and clinical populations. In addition, these stimuli appear to invoke cortical regions also associated with reading, and may be useful in examining pre-lexical reading-related networks in children as no reading of letters or words are required (Shaywitz and Shaywitz, 2005; Meschyan and Hernandez, 2006).

## Conclusion

The findings described in this study provide new data regarding developmental aspects of visual motion/form processing in children. We have identified two distinct patterns in the processing of continuously morphing, radially modulated, visual grating stimuli in children aged 5–15 years (Doucet et al., 2005; Kubova et al., 2014). VEP components revealed a lack of major changes across age for the two distinct morphological patterns, and CDR imagery identified anatomical visual cortical regions involved in the processing of complex visual stimuli. Activated visual cortical areas across age and pattern were relatively consistent, encompassing cerebellar regions as well as higher-order visual regions such as lingual gyrus, and Brodmann Areas 18, and 19.

Taken together, these results demonstrate consistent and robust VEP waveforms and visual source generators in response to apparent motion and shape change across age, VEP component, and VEP pattern. Though the visual cortical activation did not demonstrate major developmental differences, it will be of interest in the future to determine the functional significance of these two morphological patterns, including possible relationships with literacy and similar higher-order visual skills in typically developing and clinical populations.

## Author Contributions

All authors listed, have made substantial, direct and intellectual contribution to the work, and approved it for publication.

## Conflict of Interest Statement

The authors declare that the research was conducted in the absence of any commercial or financial relationships that could be construed as a potential conflict of interest.

## Supplementary Material

FIGURE S1 **Visual stimuli.** Visual stimuli for the experiment consisted of a high contrast sinusoidal concentric grating that transitioned into a radially modulated grating or circle-star pattern. Adapted from Doucet et al. (2005, 2006).
