# Supplementary figures and images for: Distinct Visual Evoked Potential Morphological Patterns for Apparent Motion Processing in School-Aged Children
Source: Front Hum Neurosci. 2016 Jun 28;10:277. doi: 10.3389/fnhum.2016.00277 (PMC4923113; doi:10.3389/fnhum.2016.00277)

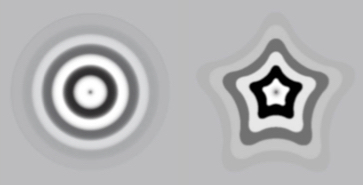

Supplement: FIGURE S1 — Visual stimuli. Visual stimuli for the experiment consisted of a high contrast sinusoidal concentric grating that transitioned into a radially modulated grating or circle-star pattern. Adapted from Doucet et al. (2005, 2006). [file Image_1.JPG]
